# Genes for endosomal NHE6 and NHE9 are misregulated in autism brains

**DOI:** 10.1038/mp.2013.28

**Published:** 2013-03-19

**Authors:** M Schwede, K Garbett, K Mirnics, D H Geschwind, E M Morrow

**Affiliations:** 1 Department of Molecular Biology, Cell Biology and Biochemistry, Institute for Brain Science, Brown University, Laboratory for Molecular Medicine, Providence, RI, USA; 2 Department of Psychiatry, Vanderbilt University, Nashville, TN, USA; 3 Vanderbilt Kennedy Center for Research on Human Development, Vanderbilt University, Nashville, TN, USA; 4 UCLA Center for Autism Research and Treatment, Semel Institute for Neuroscience and Behavior, Los Angeles, CA, USA; 5 Program in Neurogenetics, Department of Neurology, David Geffen School of Medicine at UCLA, Los Angeles, CA, USA; 6 Department of Human Genetics, David Geffen School of Medicine at UCLA, Los Angeles, CA, USA; 7 Department of Psychiatry and Human Behavior, Emma Pendleton Bradley Hospital, Alpert Medical School of Brown University, East Providence, RI, USA

Autism is a highly heterogeneous neurodevelopmental disorder with impaired language, social communication, and restricted and repetitive interests and behavior. Monogenic developmental brain disorders with autism features such as Rett syndrome, Angelman syndrome, Fragile X syndrome, and others provide important tractable models of relevance to severe autism. In addition to observing autism symptoms in the monogenic condition, if the gene responsible is also significantly misregulated in the brains of people with idiopathic autism, then these data provide substantial independent support for the importance of the gene in autism pathophysiology.

Given this logic, we set out to test if there were gene expression changes in postmortem brain tissue from patients with idiopathic autism in the genes of interest that encode the Na^+^/H^+^ exchanger family of proteins, with particular interest in the forms of exchangers localized to endosomes, namely, NHE6 and NHE9. Mutations in the X-linked endosomal *Na*^*+*^*/H*^*+*^ *Exchanger 6* (*NHE6*, also known as *SLC9A6*) represent a novel neurogenetic syndrome with variable expressivity.^1^ In a systematic, large-scale resequencing screen of X-chromosome coding exons in >200 pedigrees consistent with X-linked intellectual disability, *NHE6* was among the top six most recurrently mutated genes.^2^ The ‘Christianson syndrome', based on the initial clinical description, reported an association with autistic symptoms as has been reported subsequently.^3, 4^ In parallel to the description of autistic symptoms associated with mutations in *NHE6,* Morrow *et al.*^5^ published mutations in the highly related endosomal protein *NHE9* in severe autism with epilepsy. Endosomal processes, such as would be suggested by mutations in *NHE6* and *NHE9,* represent an important cellular mechanism for investigations regarding disorders of cognitive development. Interestingly, of the six top genes implicated in the Tarpey *et al.*^2^ study, two of the genes, *NHE6* and *AP1S2*, are known to be involved in endosomal mechanisms.

We therefore investigated whether the expression of *NHE* genes, *NHE6* and *NHE9*, were altered in autism cerebral cortex. We did this by renormalizing and analyzing publically available microarray data. Specifically, we first analyzed data from Voineagu *et al.*,^6^ which were previously used to compare autism and control cortex (*n*=29 of each). We renormalized the data as described in Voineagu *et al.*,^6^ except for when we wished to analyze a subset of *NHE* genes that were excluded from the analysis because of low expression. Among their results, Voineagu *et al.*^6^ reported that synapse-associated genes were downregulated in autism cortex,^6^ and we confirmed this result by performing differential gene expression analysis^7^ between autism and control cortex (for detailed Methods, please see Supplementary Information). We then performed DAVID functional annotation clustering analysis^8, 9^ on the 197 genes with at least 1.3-fold reduction in autism cortex and *P*<0.05 (after Benjamini-Hochberg adjustment^10^), which were the differential expression cutoffs described by Voineagu *et al.*^6^ Indeed, the most significant gene set in the top scoring cluster of the DAVID results was the Gene Ontology term ‘synapse,' for which 21 genes overlapped with those downregulated in autism cortex (BH-adjusted overlap *P*-value=2.2 × 10^−7^). This confirmed that synapse-related genes, such as *GABRA1* and *CHRM1*, were downregulated in autism cortex in the Voineagu *et al.*^6^ data set (Supplementary Tables 1 and 2 for description of the downregulated synapse genes). We next tested whether *NHE* genes (*NHE1–11*) were differentially expressed between autism and control cortex using a *t*-test (Supplementary Table 3). Notably, *NHE1* and *NHE6* were significantly downregulated in autism cortex (*P*=0.0030 and 0.0042, respectively) and *NHE9* was significantly upregulated (*P*=0.00075), yet other *NHE* genes were not significantly differentially expressed between autism and control cortex (*P*>0.15).

We further hypothesized that changes in these genes were reflective of broader changes in gene expression, such as downregulation of synapse genes. To investigate the functional changes associated with *NHE1*, *NHE6* and *NHE9* gene expression, we found Pearson correlation coefficients between each of these genes and the average expression of the 21 synapse-related genes. *NHE1* was not significantly correlated with the synapse genes (*r*=0.15, *P*=0.28), but *NHE6* and *NHE9* both were strongly correlated with the synapse genes (*r*=0.86, *P*=4.2 × 10^−18^ for *NHE6*; *r*=−0.62, *P*=2.2 × 10^−7^ for *NHE9*—see Figure 1). Furthermore, the sub-population of samples with the lowest *NHE6* expression was almost entirely autism cases, as was the sub-population of high *NHE9* expression. Additionally, *NHE6* and *NHE9* were negatively correlated (*P*=0.00015), and when *NHE6* expression was high, *NHE9* was tightly regulated. A similar decrease in *NHE9* variability could be seen with high synapse gene expression (Figure 1). Thus, a sub-population within autism is not only associated with lower synapse gene expression, but also with lower *NHE6* expression and increased and potentially misregulated *NHE9* expression. We also studied the correlation of *NHE6* and *NHE9* with the downregulated synapse gene set during normal brain development. We capitalized on the large mRNA-seq data set from human brain made available by the Allen Brain Institute (http://www.developinghumanbrain.org). We find that *NHE6* clusters strongly during development with this synapse gene group (see Supplementary Information). *NHE9* expression was far lower embryonically, increased postnatally but did not appreciably cluster with this synapse group in typical brain development (Supplementary Figures 1–3).

We used two independent microarray data sets for autism and control cerebral cortex to validate both the differential gene expression of *NHE6* and *NHE9* in autism cases and these genes' associations with synapse genes. The validation data sets were from Chow *et al.*^11^ (*n*=15 autism, *n*=18 control) and Garbett *et al.*^12^ (*n*=6 of each; data set details in Supplementary Table 4). The Chow *et al.*^11^ data were downloaded from GEO (GSE28475), and the Garbett *et al.*^12^ data were provided by the authors. In the Garbett *et al.*^12^ data, *NHE9* was significantly higher in autism than control cortex (*P*=0.039), and while *NHE6* was lower on average in autism cortex, this was not statistically significant (*P*=0.22), although this may have been due to low sample size (Figure 1). In the Chow *et al.*^11^ data, although *NHE6* and *NHE9* had lower and higher expression in autism cortex compared with control, respectively, these trends were not significant (*P*=0.38 and 0.17). However, the mean expression of synapse genes was also not significantly different in the Chow *et al.*^11^ data set (*P*=0.46), suggesting that the Voineagu *et al.*^6^ and Chow *et al.*^11^ data sets represent different populations. Additionally, *NHE6* and *NHE9* were negatively correlated across data sets, except for in Garbett *et al.*,^12^ in which the correlation was not significant (*P*=0.56) but suggesting as yet unknown mechanisms of interaction. Despite this lack of significance in the independent data sets, these genes' associations with synapse genes remained strong in both additional data sets: *NHE6* is positively associated with synapse genes (*r*=0.59, *P*=0.00029 for *NHE6* in Chow *et al.*^11^ study; and *r*=0.6, *P*=0.041 for *NHE6* in Garbett *et al.*^12^ study—see Figure 1) and *NHE9* negatively associated (*r*=−0.72, *P*=2.0 × 10^−6^ for *NHE9* in Chow *et al.*^11^ study; and *r*=−0.78, *P*=0.0029 for *NHE9* in Garbett *et al.*^12^ study—see Figure 1). Finally, we tested for an association of gene expression for *NHE6* and *NHE9* and well-established autism-related genes such as *SHANK2/3*, *NLGN4X*, *NRXN1* and *PTEN*. Notably, *NHE6* and *NHE9* each showed a strong association with *NRXN1* (*P*=2 × 10^−10^ for *NHE6*, and *P*=2.9 × 10^−8^ for *NHE9*; Supplementary Figures 4 and 5).

In summary, we find interesting gene expression changes in endosomal *NHE6* and *NHE9* in postmortem autism brains. These gene expression changes are largely replicated across data sets or the trends of these changes are maintained given limitations in sample size. We also report a strong correlation of endosomal *NHE6* and *NHE9* gene expression with the synapse genes across all data sets. The strong correlation of endosomal NHEs with synapse genes suggests that changes in synapse genes in autism involves a cellular mechanism that involves endosomal NHE6 and NHE9 in at least some autism brains. In conclusion, these gene expression studies in postmortem brains from patients with idiopathic autism provide additional support, in addition to the association of autism symptoms with the single-gene mutations, that endosomal NHEs are mechanistically involved in the pathophysiology of autism.

## Figures and Tables

**Figure 1:**
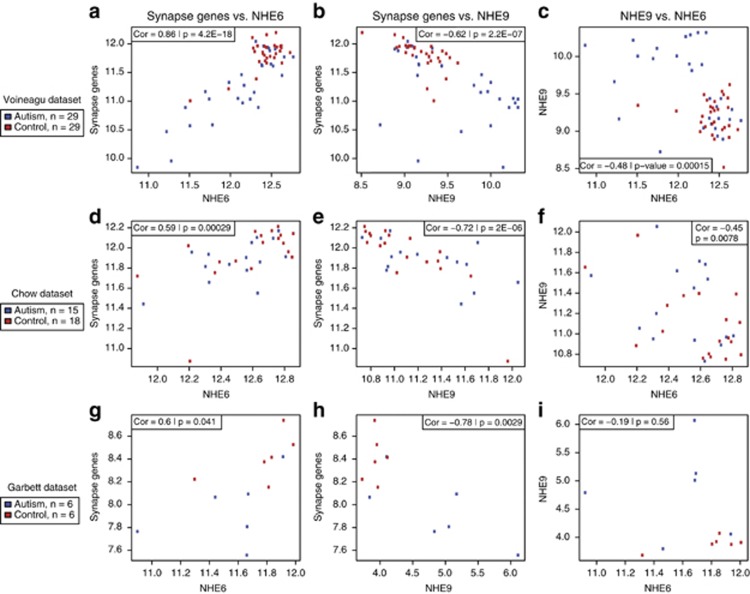
Expression plots of *NHE6* versus synapse genes, *NHE9* versus synapse genes and *NHE6* versus *NHE9* in three independent data sets. We compared the average log base 2 expression of 21 synapse genes with that of *NHE6* and *NHE9* expression in microarray data sets published by Voineagu *et al.*^6^ (**a**–**c**, *n*=58), Chow *et al.*^11^ (**d**–**f**, *n*=33) and Garbett *et al.*^12^ (**g**–**i**, *n*=12). Each data set was designed to compare gene expression in autism and control cerebral cortex. The synapse genes used were the 21 synapse genes downregulated in autism cortex compared with control in the Voineagu *et al.*^6^ data set (Supplementary Tables 1 and 2).
