# Supplementary figures and images for: Genes for endosomal NHE6 and NHE9 are misregulated in autism brains
Source: Mol Psychiatry. 2013 Mar 19;19(3):277–9. doi: 10.1038/mp.2013.28 (PMC3932404; doi:10.1038/mp.2013.28)

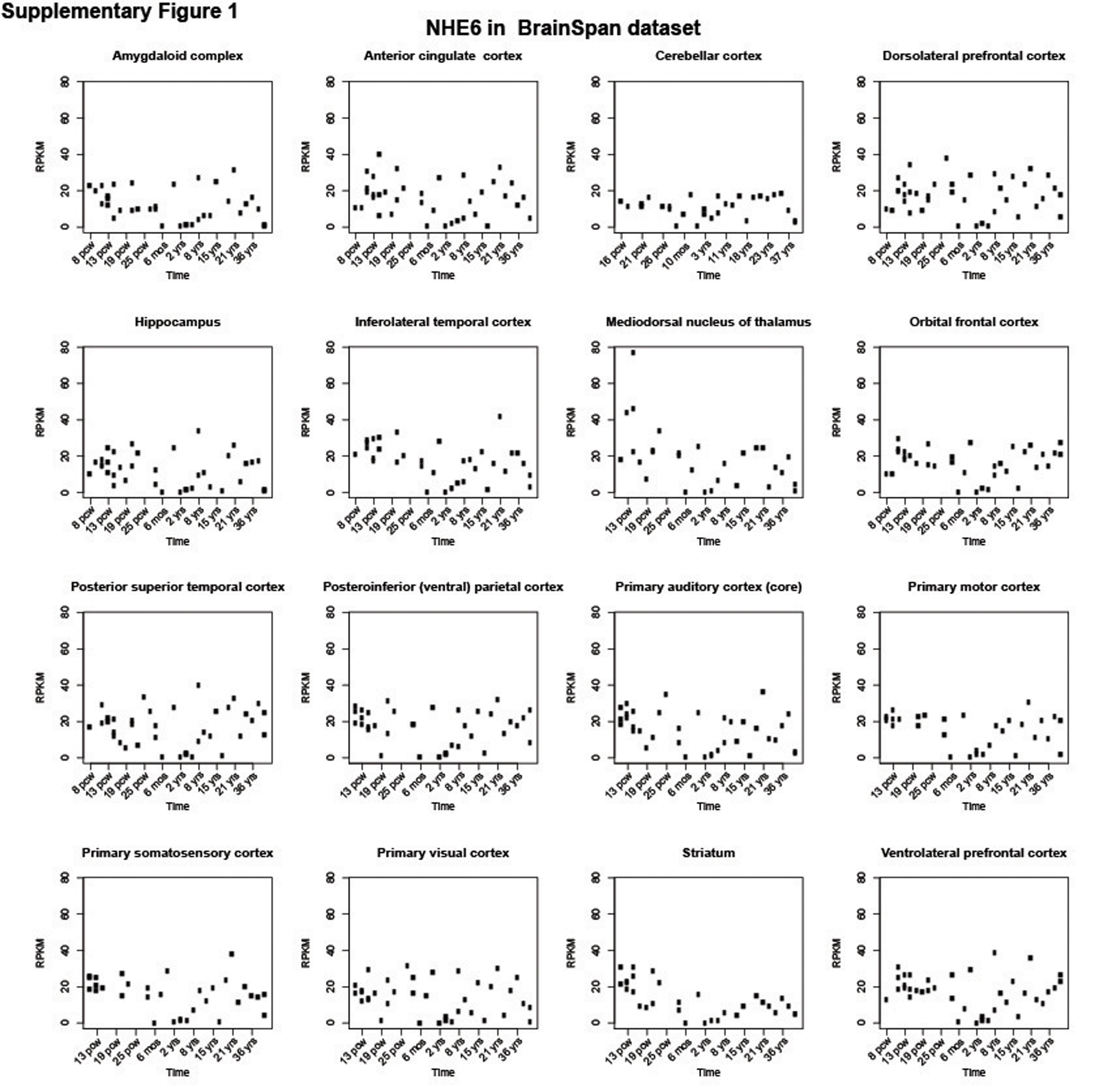

Supplement: Supplementary Figure 1 [file mp201328x1.tif]

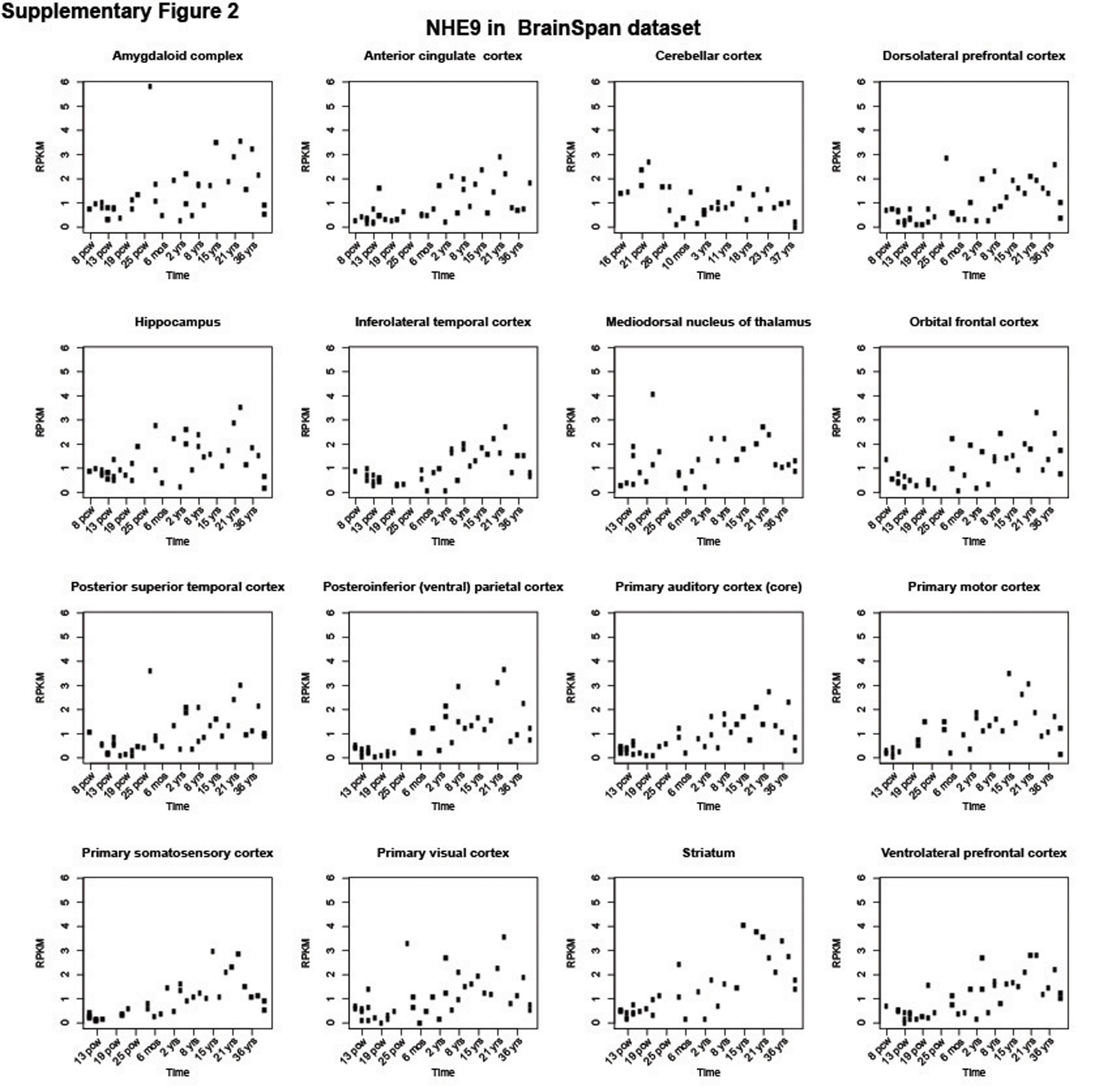

Supplement: Supplementary Figure 2 [file mp201328x2.tif]

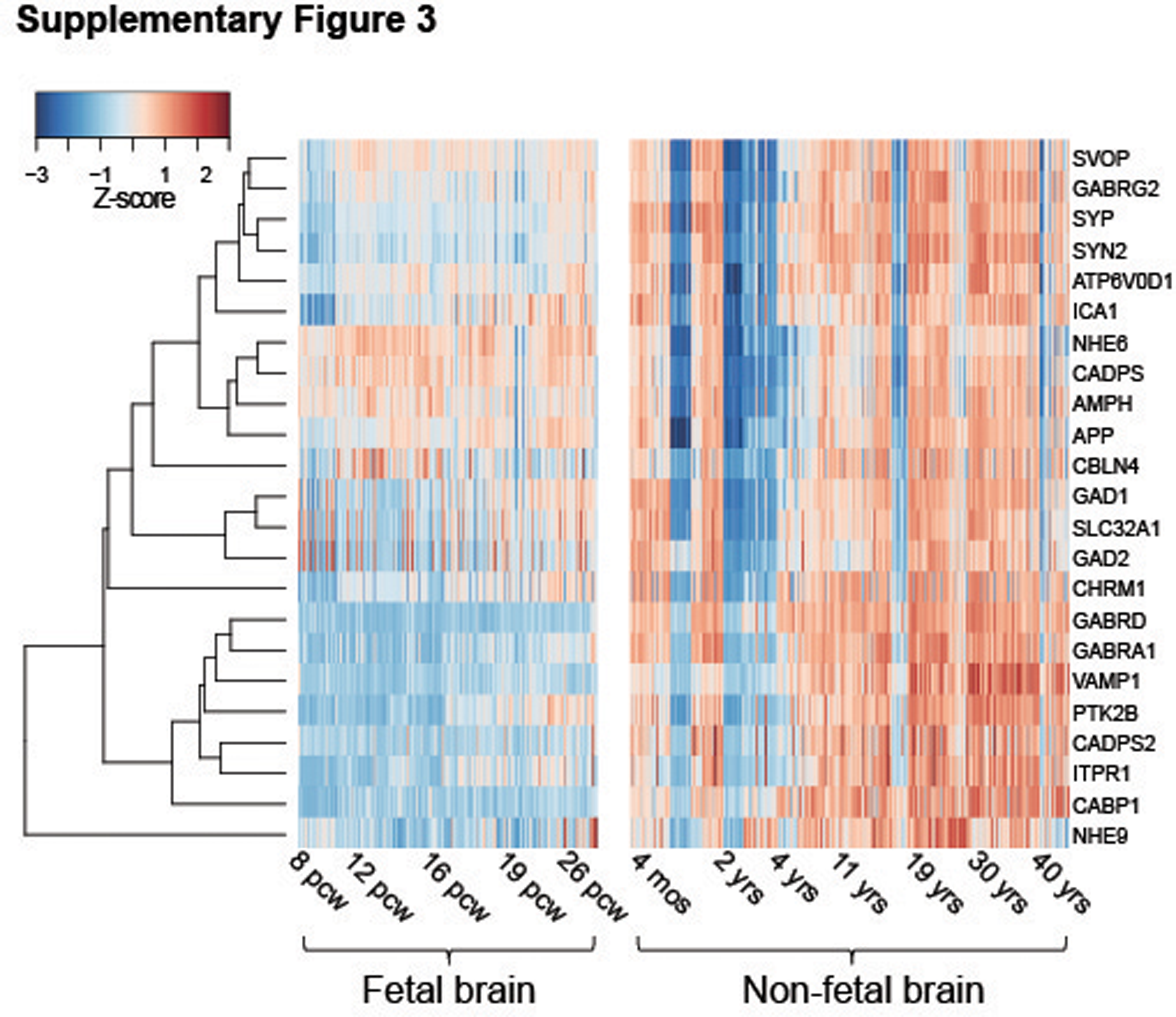

Supplement: Supplementary Figure 3 [file mp201328x3.tif]

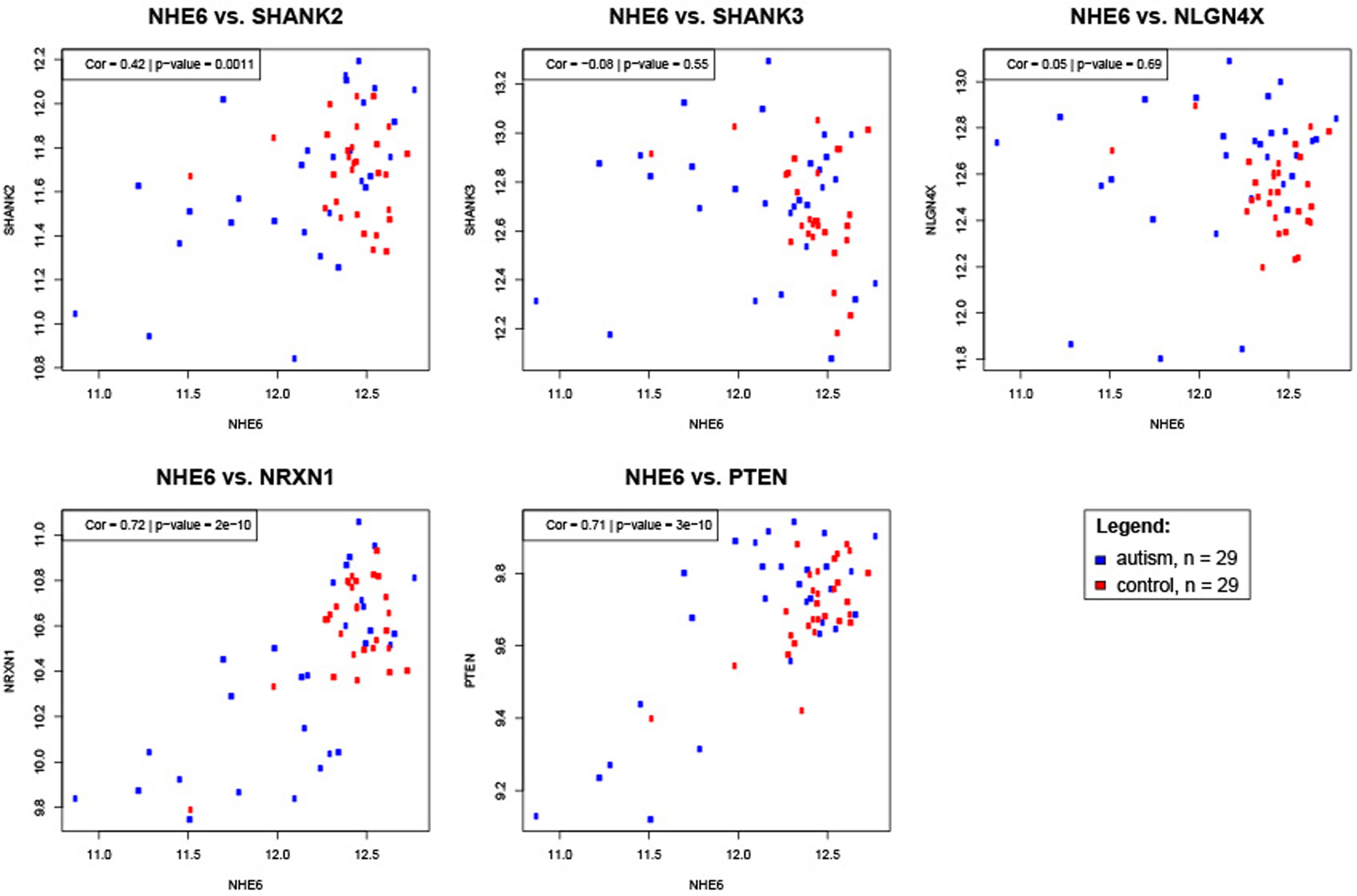

Supplement: Supplementary Figure 4 [file mp201328x4.tif]

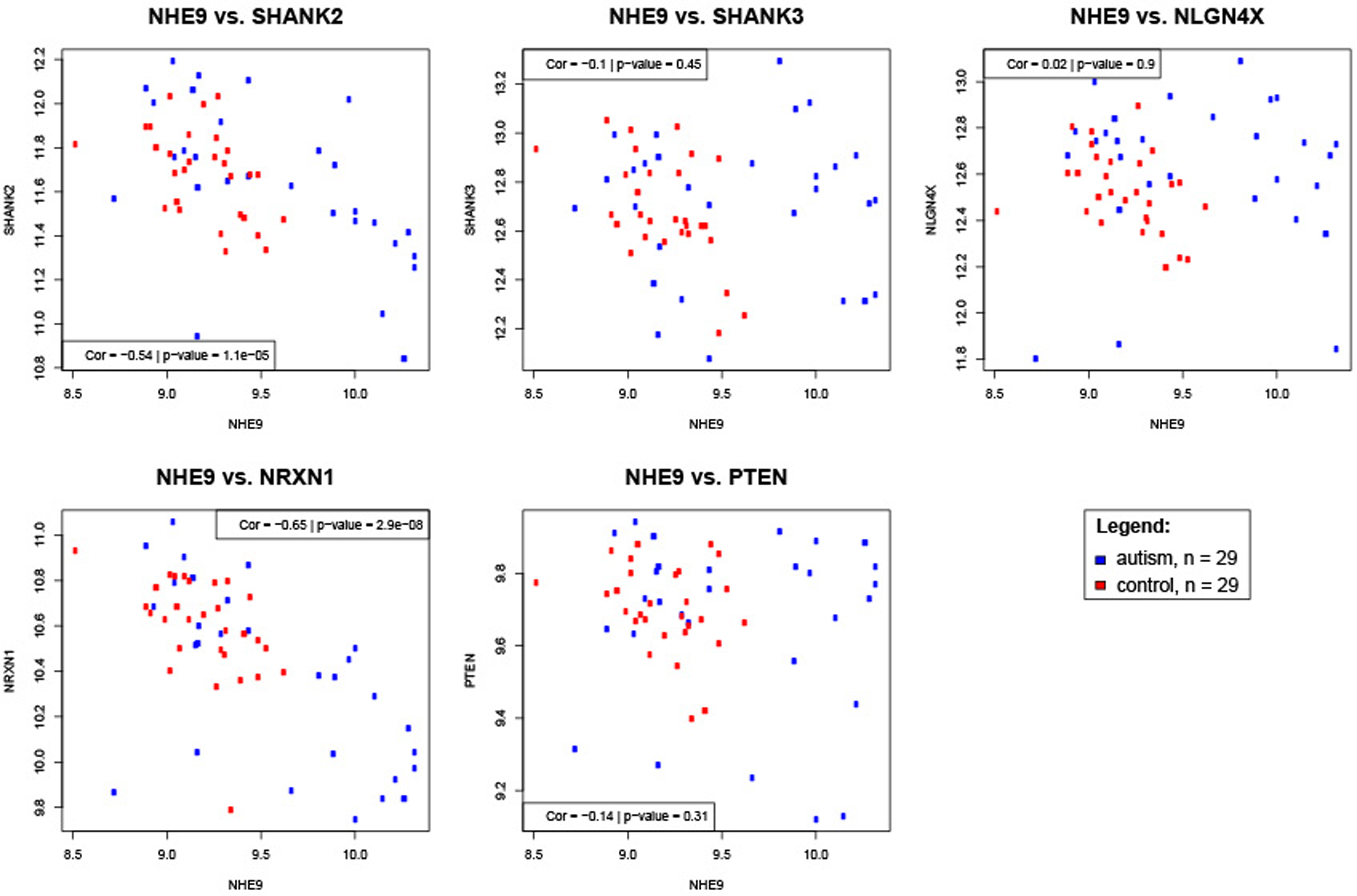

Supplement: Supplementary Figure 5 [file mp201328x5.tif]
