# Supplementary material for: Genes for endosomal NHE6 and NHE9 are misregulated in autism brains
Source: Mol Psychiatry. 2013 Mar 19;19(3):277–9. doi: 10.1038/mp.2013.28 (PMC3932404; doi:10.1038/mp.2013.28)
Supplement: Supplementary Table 1 [file mp201328x6.doc]

**Supplementary Table 1.** Genome-wide re-analysis identified 21 synapse genes to be down-regulated.

| Gene | P-value | Fold change in autism |
| --- | --- | --- |
| AMPH | 0.029 | 0.67 |
| APP | 0.047 | 0.77 |
| ATP6V0D1 | 0.017 | 0.75 |
| CABP1 | 0.0037 | 0.72 |
| CADPS | 0.00050 | 0.72 |
| CADPS2 | 0.0015 | 0.65 |
| CBLN4 | 0.047 | 0.72 |
| CHRM1 | 0.0017 | 0.72 |
| GABRA1 | 0.023 | 0.63 |
| GABRD | 0.0063 | 0.77 |
| GABRG2 | 0.024 | 0.62 |
| GAD1 | 0.010 | 0.58 |
| GAD2 | 0.012 | 0.55 |
| ICA1 | 0.00065 | 0.72 |
| ITPR1 | 0.019 | 0.72 |
| PTK2B | 0.017 | 0.73 |
| SLC32A1 | 0.032 | 0.58 |
| SVOP | 0.0089 | 0.70 |
| SYN2 | 0.032 | 0.76 |
| SYP | 0.017 | 0.76 |
| VAMP1 | 0.00074 | 0.54 |
