# Supplementary material for: Genes for endosomal NHE6 and NHE9 are misregulated in autism brains
Source: Mol Psychiatry. 2013 Mar 19;19(3):277–9. doi: 10.1038/mp.2013.28 (PMC3932404; doi:10.1038/mp.2013.28)
Supplement: Supplementary Table 2 [file mp201328x7.doc]

Supplementary Table 2

| Databse Object Symbol | Gene Ontology ID | Gene Ontology Term | Evidence code | Evidence description | DB Object Name | DB Object Synonym (|Synonym) | DB Object Type | Taxon ID | Species | Assigned by |
| --- | --- | --- | --- | --- | --- | --- | --- | --- | --- | --- |
| AMPH | GO:0030672 | synaptic vesicle membrane | IEA | Inferred from Electronic Annotation | Amphiphysin | AMPH|AMPH_CHICK | protein | taxon:9031 | Gallus gallus | UniProtKB |
| AMPH | GO:0030672 | synaptic vesicle membrane | IEA | Inferred from Electronic Annotation | Amphiphysin | AMPH|AMPH1|AMPH_HUMAN | protein | taxon:9606 | Homo sapiens | UniProtKB |
| Amph | GO:0030672 | synaptic vesicle membrane | IEA | Inferred from Electronic Annotation | amphiphysin |  | gene | taxon:10116 | Rattus norvegicus | UniProtKB |
| Amph | GO:0045202 | synapse | IDA | Inferred from Direct Assay | Amphiphysin | Amp|amph|amphiphysin|CG8604|DAMP|dAmph | protein | taxon:7227 | Drosophila melanogaster | FlyBase |
| Amph | GO:0045202 | synapse | IDA | Inferred from Direct Assay | Amphiphysin | Amp|amph|amphiphysin|CG8604|DAMP|dAmph | protein | taxon:7227 | Drosophila melanogaster | FlyBase |
| Amph | GO:0045202 | synapse | IEA | Inferred from Electronic Annotation | amphiphysin |  | protein | taxon:10090 | Mus musculus | UniProtKB |
| AMPH | GO:0008021 | synaptic vesicle | IEA | Inferred from Electronic Annotation | Amphiphysin | AMPH|F1NZ03_CHICK | protein | taxon:9031 | Gallus gallus | ENSEMBL |
| AMPH | GO:0008021 | synaptic vesicle | IEA | Inferred from Electronic Annotation | AMPH protein | A5D783_BOVIN|AMPH|Bt.89279 | protein | taxon:9913 | Bos taurus | ENSEMBL |
| AMPH | GO:0008021 | synaptic vesicle | TAS | Traceable Author Statement | Amphiphysin | AMPH|AMPH1|AMPH_HUMAN | protein | taxon:9606 | Homo sapiens | PINC |
| Amph | GO:0008021 | synaptic vesicle | IDA | Inferred from Direct Assay | amphiphysin |  | protein | taxon:10090 | Mus musculus | MGI |
| Amph | GO:0008021 | synaptic vesicle | IEA | Inferred from Electronic Annotation | amphiphysin |  | gene | taxon:10116 | Rattus norvegicus | ENSEMBL |
| Amph | GO:0008021 | synaptic vesicle | ISO | Inferred from Sequence Orthology | amphiphysin |  | gene | taxon:10116 | Rattus norvegicus | RGD |
| APP | GO:0031594 | neuromuscular junction | IEA | Inferred from Electronic Annotation | Uncharacterized protein | APP|E1C440_CHICK | protein | taxon:9031 | Gallus gallus | ENSEMBL |
| APP | GO:0031594 | neuromuscular junction | IEA | Inferred from Electronic Annotation | Uncharacterized protein | APP|F1NTQ6_CHICK | protein | taxon:9031 | Gallus gallus | ENSEMBL |
| APP | GO:0031594 | neuromuscular junction | IEA | Inferred from Electronic Annotation | Amyloid beta (A4) protein | APP|Bt.34436|Q08E54_BOVIN | protein | taxon:9913 | Bos taurus | ENSEMBL |
| APP | GO:0031594 | neuromuscular junction | IEA | Inferred from Electronic Annotation | Amyloid beta A4 protein | A4_BOVIN|APP | protein | taxon:9913 | Bos taurus | ENSEMBL |
| APP | GO:0031594 | neuromuscular junction | IEA | Inferred from Electronic Annotation | Gamma-secretase C-terminal fragment 59 | APP|F1P603_CANFA | protein | taxon:9615 | Canis familiaris | ENSEMBL |
| APP | GO:0031594 | neuromuscular junction | IEA | Inferred from Electronic Annotation | Amyloid beta A4 protein | A4_CANFA|APP | protein | taxon:9615 | Canis familiaris | ENSEMBL |
| APP | GO:0031594 | neuromuscular junction | IEA | Inferred from Electronic Annotation | Amyloid beta A4 protein | A4|A4_HUMAN|AD1|APP | protein | taxon:9606 | Homo sapiens | ENSEMBL |
| APP | GO:0031594 | neuromuscular junction | IEA | Inferred from Electronic Annotation | Amyloid protein variant 2 | APP|LOC100522215|Q2XQ99_PIG | protein | taxon:9823 | Sus scrofa | ENSEMBL |
| App | GO:0031594 | neuromuscular junction | IDA | Inferred from Direct Assay | amyloid beta (A4) precursor protein | Abeta|Adap|appican|betaAPP|Cvap|protease nexin II | protein | taxon:10090 | Mus musculus | MGI |
| App | GO:0031594 | neuromuscular junction | IEA | Inferred from Electronic Annotation | amyloid beta (A4) precursor protein |  | gene | taxon:10116 | Rattus norvegicus | ENSEMBL |
| App | GO:0031594 | neuromuscular junction | ISO | Inferred from Sequence Orthology | amyloid beta (A4) precursor protein |  | gene | taxon:10116 | Rattus norvegicus | RGD |
| APP | GO:0045202 | synapse | IDA | Inferred from Direct Assay | Amyloid beta A4 protein | A4|A4_HUMAN|AD1|APP | protein | taxon:9606 | Homo sapiens | MGI |
| App | GO:0045202 | synapse | ISO | Inferred from Sequence Orthology | amyloid beta (A4) precursor protein |  | gene | taxon:10116 | Rattus norvegicus | RGD |
| ATP6V0D1 | GO:0008021 | synaptic vesicle | IEA | Inferred from Electronic Annotation | V-type proton ATPase subunit d 1 | ATP6V0D1|F8WEN9_HUMAN | protein | taxon:9606 | Homo sapiens | ENSEMBL |
| ATP6V0D1 | GO:0008021 | synaptic vesicle | IEA | Inferred from Electronic Annotation | V-type proton ATPase subunit d 1 | ATP6V0D1|H3BPS0_HUMAN | protein | taxon:9606 | Homo sapiens | ENSEMBL |
| ATP6V0D1 | GO:0008021 | synaptic vesicle | IEA | Inferred from Electronic Annotation | V-type proton ATPase subunit d 1 | ATP6V0D1|H3BTB4_HUMAN | protein | taxon:9606 | Homo sapiens | ENSEMBL |
| ATP6V0D1 | GO:0008021 | synaptic vesicle | IEA | Inferred from Electronic Annotation | V-type proton ATPase subunit d 1 | ATP6D|ATP6V0D1|VA0D1_HUMAN|VPATPD | protein | taxon:9606 | Homo sapiens | ENSEMBL |
| Atp6v0d1 | GO:0008021 | synaptic vesicle | ISO | Inferred from Sequence Orthology | ATPase, H+ transporting, lysosomal V0 subunit D1 | Ac39|Atp6d|lysosomal 38kDa|P39|Vma6 | protein | taxon:10090 | Mus musculus | RGD |
| Atp6v0d1 | GO:0008021 | synaptic vesicle | IDA | Inferred from Direct Assay | ATPase, H+ transporting, lysosomal V0 subunit D1 |  | gene | taxon:10116 | Rattus norvegicus | RGD |
| CABP1 | GO:0045211 | postsynaptic membrane | IEA | Inferred from Electronic Annotation | Calcium-binding protein 1 | CABP1|CABP1_BOVIN | protein | taxon:9913 | Bos taurus | UniProtKB |
| CABP1 | GO:0045211 | postsynaptic membrane | IEA | Inferred from Electronic Annotation | Calcium-binding protein 1 | CABP1|CABP1_HUMAN | protein | taxon:9606 | Homo sapiens | UniProtKB |
| CABP1 | GO:0014069 | postsynaptic density | IEA | Inferred from Electronic Annotation | Uncharacterized protein | CABP1|F1P428_CHICK | protein | taxon:9031 | Gallus gallus | ENSEMBL |
| CABP1 | GO:0014069 | postsynaptic density | ISS | Inferred from Sequence or Structural Similarity | Calcium-binding protein 1 | CABP1|CABP1_BOVIN | protein | taxon:9913 | Bos taurus | AgBase |
| CABP1 | GO:0014069 | postsynaptic density | IDA | Inferred from Direct Assay | Calcium-binding protein 1 | CABP1|CABP1_HUMAN | protein | taxon:9606 | Homo sapiens | UniProtKB |
| CABP1 | GO:0014069 | postsynaptic density | IEA | Inferred from Electronic Annotation | Uncharacterized protein | CABP1|F1RJI3_PIG | protein | taxon:9823 | Sus scrofa | ENSEMBL |
| Cabp1 | GO:0014069 | postsynaptic density | ISO | Inferred from Sequence Orthology | calcium binding protein 1 | caldendrin | protein | taxon:10090 | Mus musculus | MGI |
| Cabp1 | GO:0014069 | postsynaptic density | IDA | Inferred from Direct Assay | calcium binding protein 1 |  | gene | taxon:10116 | Rattus norvegicus | UniProtKB |
| CADPS | GO:0045202 | synapse | IEA | Inferred from Electronic Annotation | Calcium-dependent secretion activator 1 | CADPS|CAPS|CAPS1|CAPS1_HUMAN|KIAA1121 | protein | taxon:9606 | Homo sapiens | UniProtKB |
| Cadps | GO:0045202 | synapse | IEA | Inferred from Electronic Annotation | Ca2+-dependent secretion activator | CAPS1 | protein | taxon:10090 | Mus musculus | UniProtKB |
| Cadps | GO:0045202 | synapse | IEA | Inferred from Electronic Annotation | Ca++-dependent secretion activator |  | gene | taxon:10116 | Rattus norvegicus | UniProtKB |
| CADPS2 | GO:0042734 | presynaptic membrane | IEA | Inferred from Electronic Annotation | Uncharacterized protein | CADPS2|F1NKU1_CHICK | protein | taxon:9031 | Gallus gallus | ENSEMBL |
| CADPS2 | GO:0042734 | presynaptic membrane | IEA | Inferred from Electronic Annotation | Uncharacterized protein | A7MB25_BOVIN|Bt.91583|CADPS2 | protein | taxon:9913 | Bos taurus | ENSEMBL |
| CADPS2 | GO:0042734 | presynaptic membrane | IEA | Inferred from Electronic Annotation | Calcium-dependent secretion activator 2 | CADPS2|CAPS2|CAPS2_HUMAN|KIAA1591 | protein | taxon:9606 | Homo sapiens | ENSEMBL |
| CADPS2 | GO:0042734 | presynaptic membrane | IEA | Inferred from Electronic Annotation | Uncharacterized protein | CADPS2|F1SLX7_PIG | protein | taxon:9823 | Sus scrofa | ENSEMBL |
| Cadps2 | GO:0042734 | presynaptic membrane | IDA | Inferred from Direct Assay | Ca2+-dependent activator protein for secretion 2 | Caps2|cpd2 | protein | taxon:10090 | Mus musculus | MGI |
| Cadps2 | GO:0042734 | presynaptic membrane | ISO | Inferred from Sequence Orthology | Ca++-dependent secretion activator 2 |  | gene | taxon:10116 | Rattus norvegicus | RGD |
| Cadps2 | GO:0042734 | presynaptic membrane | IEA | Inferred from Electronic Annotation | Protein Cadps2 | Cadps2|F1M0K0_RAT | protein | taxon:10116 | Rattus norvegicus | ENSEMBL |
| Cadps2 | GO:0045202 | synapse | IEA | Inferred from Electronic Annotation | Ca2+-dependent activator protein for secretion 2 | Caps2|cpd2 | protein | taxon:10090 | Mus musculus | UniProtKB |
| CADPS2 | GO:0045211 | postsynaptic membrane | IEA | Inferred from Electronic Annotation | Uncharacterized protein | CADPS2|F1NKU1_CHICK | protein | taxon:9031 | Gallus gallus | ENSEMBL |
| CADPS2 | GO:0045211 | postsynaptic membrane | IEA | Inferred from Electronic Annotation | Uncharacterized protein | A7MB25_BOVIN|Bt.91583|CADPS2 | protein | taxon:9913 | Bos taurus | ENSEMBL |
| CADPS2 | GO:0045211 | postsynaptic membrane | IEA | Inferred from Electronic Annotation | Calcium-dependent secretion activator 2 | CADPS2|CAPS2|CAPS2_HUMAN|KIAA1591 | protein | taxon:9606 | Homo sapiens | ENSEMBL |
| CADPS2 | GO:0045211 | postsynaptic membrane | IEA | Inferred from Electronic Annotation | Uncharacterized protein | CADPS2|F1SLX7_PIG | protein | taxon:9823 | Sus scrofa | ENSEMBL |
| Cadps2 | GO:0045211 | postsynaptic membrane | IDA | Inferred from Direct Assay | Ca2+-dependent activator protein for secretion 2 | Caps2|cpd2 | protein | taxon:10090 | Mus musculus | MGI |
| Cadps2 | GO:0045211 | postsynaptic membrane | ISO | Inferred from Sequence Orthology | Ca++-dependent secretion activator 2 |  | gene | taxon:10116 | Rattus norvegicus | RGD |
| Cadps2 | GO:0045211 | postsynaptic membrane | IEA | Inferred from Electronic Annotation | Protein Cadps2 | Cadps2|F1M0K0_RAT | protein | taxon:10116 | Rattus norvegicus | ENSEMBL |
| Cadps2 | GO:0008021 | synaptic vesicle | IDA | Inferred from Direct Assay | Ca2+-dependent activator protein for secretion 2 | Caps2|cpd2 | protein | taxon:10090 | Mus musculus | MGI |
| Cadps2 | GO:0008021 | synaptic vesicle | ISO | Inferred from Sequence Orthology | Ca++-dependent secretion activator 2 |  | gene | taxon:10116 | Rattus norvegicus | RGD |
| CBLN4 | GO:0045202 | synapse | IEA | Inferred from Electronic Annotation | Cerebellin-4 | CBLN4|CBLN4_HUMAN|CBLNL1|UNQ718/PRO1382 | protein | taxon:9606 | Homo sapiens | UniProtKB |
| Cbln4 | GO:0045202 | synapse | IEA | Inferred from Electronic Annotation | cerebellin 4 precursor protein |  | protein | taxon:10090 | Mus musculus | UniProtKB |
| CHRM1 | GO:0032279 | asymmetric synapse | IEA | Inferred from Electronic Annotation | Muscarinic acetylcholine receptor M1 | CHRM1|F5GZF8_HUMAN | protein | taxon:9606 | Homo sapiens | ENSEMBL |
| CHRM1 | GO:0032279 | asymmetric synapse | IEA | Inferred from Electronic Annotation | Muscarinic acetylcholine receptor M1 | ACM1_HUMAN|CHRM1 | protein | taxon:9606 | Homo sapiens | ENSEMBL |
| CHRM1 | GO:0032279 | asymmetric synapse | IEA | Inferred from Electronic Annotation | Muscarinic acetylcholine receptor M1 | CHRM1|Q96RH1_HUMAN | protein | taxon:9606 | Homo sapiens | ENSEMBL |
| Chrm1 | GO:0032279 | asymmetric synapse | ISO | Inferred from Sequence Orthology | cholinergic receptor, muscarinic 1, CNS | AW495047|Chrm-1|M1|M1R|muscarinic acetylcholine receptor 1 | protein | taxon:10090 | Mus musculus | RGD |
| Chrm1 | GO:0032279 | asymmetric synapse | IDA | Inferred from Direct Assay | cholinergic receptor, muscarinic 1 |  | gene | taxon:10116 | Rattus norvegicus | RGD |
| Chrm1 | GO:0045202 | synapse | IEA | Inferred from Electronic Annotation | cholinergic receptor, muscarinic 1, CNS | AW495047|Chrm-1|M1|M1R|muscarinic acetylcholine receptor 1 | protein | taxon:10090 | Mus musculus | UniProtKB |
| Chrm1 | GO:0045202 | synapse | TAS | Traceable Author Statement | cholinergic receptor, muscarinic 1 |  | gene | taxon:10116 | Rattus norvegicus | RGD |
| CHRM1 | GO:0045211 | postsynaptic membrane | IEA | Inferred from Electronic Annotation | Muscarinic acetylcholine receptor M1 | ACM1_HUMAN|CHRM1 | protein | taxon:9606 | Homo sapiens | UniProtKB |
| CHRM1 | GO:0045211 | postsynaptic membrane | IEA | Inferred from Electronic Annotation | Muscarinic acetylcholine receptor M1 | ACM1_PIG|CHRM1 | protein | taxon:9823 | Sus scrofa | UniProtKB |
| Chrm1 | GO:0045211 | postsynaptic membrane | IEA | Inferred from Electronic Annotation | cholinergic receptor, muscarinic 1, CNS | AW495047|Chrm-1|M1|M1R|muscarinic acetylcholine receptor 1 | protein | taxon:10090 | Mus musculus | UniProtKB |
| Chrm1 | GO:0045211 | postsynaptic membrane | IEA | Inferred from Electronic Annotation | cholinergic receptor, muscarinic 1 |  | gene | taxon:10116 | Rattus norvegicus | UniProtKB |
| CHRM1 | GO:0014069 | postsynaptic density | IEA | Inferred from Electronic Annotation | Muscarinic acetylcholine receptor M1 | CHRM1|F5GZF8_HUMAN | protein | taxon:9606 | Homo sapiens | ENSEMBL |
| CHRM1 | GO:0014069 | postsynaptic density | IEA | Inferred from Electronic Annotation | Muscarinic acetylcholine receptor M1 | ACM1_HUMAN|CHRM1 | protein | taxon:9606 | Homo sapiens | ENSEMBL |
| CHRM1 | GO:0014069 | postsynaptic density | IEA | Inferred from Electronic Annotation | Muscarinic acetylcholine receptor M1 | CHRM1|Q96RH1_HUMAN | protein | taxon:9606 | Homo sapiens | ENSEMBL |
| Chrm1 | GO:0014069 | postsynaptic density | ISO | Inferred from Sequence Orthology | cholinergic receptor, muscarinic 1, CNS | AW495047|Chrm-1|M1|M1R|muscarinic acetylcholine receptor 1 | protein | taxon:10090 | Mus musculus | RGD |
| Chrm1 | GO:0014069 | postsynaptic density | IDA | Inferred from Direct Assay | cholinergic receptor, muscarinic 1 |  | gene | taxon:10116 | Rattus norvegicus | RGD |
| Gabra1 | GO:0045202 | synapse | IEA | Inferred from Electronic Annotation | gamma-aminobutyric acid (GABA) A receptor, subunit alpha 1 | GABAA alpha 1|Gabra-1 | protein | taxon:10090 | Mus musculus | UniProtKB |
| gabra1 | GO:0045202 | synapse | IEA | Inferred from Electronic Annotation | gamma-aminobutyric acid (GABA) A receptor, alpha 1 |  | gene_product | taxon:7955 | Danio rerio | ZFIN |
| GABRA1 | GO:0045211 | postsynaptic membrane | IEA | Inferred from Electronic Annotation | Gamma-aminobutyric acid receptor subunit alpha-1 | GABRA1|GBRA1_CHICK | protein | taxon:9031 | Gallus gallus | UniProtKB |
| GABRA1 | GO:0045211 | postsynaptic membrane | IEA | Inferred from Electronic Annotation | Gamma-aminobutyric acid receptor subunit alpha-1 | GABRA1|GBRA1_BOVIN | protein | taxon:9913 | Bos taurus | UniProtKB |
| GABRA1 | GO:0045211 | postsynaptic membrane | IEA | Inferred from Electronic Annotation | Uncharacterized protein | E2RSP8_CANFA|GABRA1 | protein | taxon:9615 | Canis familiaris | UniProtKB |
| GABRA1 | GO:0045211 | postsynaptic membrane | IEA | Inferred from Electronic Annotation | Gamma-aminobutyric acid receptor subunit alpha-1 | GABRA1|GBRA1_HUMAN | protein | taxon:9606 | Homo sapiens | UniProtKB |
| GABRA1 | GO:0045211 | postsynaptic membrane | IEA | Inferred from Electronic Annotation | Uncharacterized protein | F1RR71_PIG|GABRA1 | protein | taxon:9823 | Sus scrofa | UniProtKB |
| Gabra1 | GO:0045211 | postsynaptic membrane | IEA | Inferred from Electronic Annotation | gamma-aminobutyric acid (GABA) A receptor, subunit alpha 1 | GABAA alpha 1|Gabra-1 | protein | taxon:10090 | Mus musculus | UniProtKB |
| Gabra1 | GO:0045211 | postsynaptic membrane | IEA | Inferred from Electronic Annotation | gamma-aminobutyric acid (GABA) A receptor, subunit alpha 1 | GABAA alpha 1|Gabra-1 | protein | taxon:10090 | Mus musculus | UniProtKB |
| Gabra1 | GO:0045211 | postsynaptic membrane | IEA | Inferred from Electronic Annotation | gamma-aminobutyric acid (GABA) A receptor, alpha 1 |  | gene | taxon:10116 | Rattus norvegicus | UniProtKB |
| gabra1 | GO:0045211 | postsynaptic membrane | IEA | Inferred from Electronic Annotation | gamma-aminobutyric acid (GABA) A receptor, alpha 1 |  | gene_product | taxon:7955 | Danio rerio | ZFIN |
| gabra1 | GO:0045211 | postsynaptic membrane | IEA | Inferred from Electronic Annotation | gamma-aminobutyric acid (GABA) A receptor, alpha 1 |  | gene_product | taxon:7955 | Danio rerio | ZFIN |
| Gabrd | GO:0045202 | synapse | IEA | Inferred from Electronic Annotation | gamma-aminobutyric acid (GABA) A receptor, subunit delta |  | protein | taxon:10090 | Mus musculus | UniProtKB |
| gabrd | GO:0045202 | synapse | IEA | Inferred from Electronic Annotation | gamma-aminobutyric acid (GABA) A receptor, delta |  | gene_product | taxon:7955 | Danio rerio | ZFIN |
| GABRD | GO:0045211 | postsynaptic membrane | IEA | Inferred from Electronic Annotation | Uncharacterized protein | E1BQL4_CHICK|GABRD | protein | taxon:9031 | Gallus gallus | UniProtKB |
| GABRD | GO:0045211 | postsynaptic membrane | IEA | Inferred from Electronic Annotation | Uncharacterized protein | A2VE38_BOVIN|Bt.25568|GABRD | protein | taxon:9913 | Bos taurus | UniProtKB |
| GABRD | GO:0045211 | postsynaptic membrane | IEA | Inferred from Electronic Annotation | Uncharacterized protein | E2R3M6_CANFA|GABRD | protein | taxon:9615 | Canis familiaris | UniProtKB |
| GABRD | GO:0045211 | postsynaptic membrane | IEA | Inferred from Electronic Annotation | Uncharacterized protein | F1Q344_CANFA|GABRD | protein | taxon:9615 | Canis familiaris | UniProtKB |
| GABRD | GO:0045211 | postsynaptic membrane | IEA | Inferred from Electronic Annotation | Gamma-aminobutyric acid receptor subunit delta | GABRD|GBRD_HUMAN | protein | taxon:9606 | Homo sapiens | UniProtKB |
| Gabrd | GO:0045211 | postsynaptic membrane | IEA | Inferred from Electronic Annotation | gamma-aminobutyric acid (GABA) A receptor, subunit delta |  | protein | taxon:10090 | Mus musculus | UniProtKB |
| Gabrd | GO:0045211 | postsynaptic membrane | IEA | Inferred from Electronic Annotation | gamma-aminobutyric acid (GABA) A receptor, subunit delta |  | protein | taxon:10090 | Mus musculus | UniProtKB |
| Gabrd | GO:0045211 | postsynaptic membrane | IEA | Inferred from Electronic Annotation | gamma-aminobutyric acid (GABA) A receptor, delta |  | gene | taxon:10116 | Rattus norvegicus | UniProtKB |
| gabrd | GO:0045211 | postsynaptic membrane | IEA | Inferred from Electronic Annotation | gamma-aminobutyric acid (GABA) A receptor, delta |  | gene_product | taxon:7955 | Danio rerio | ZFIN |
| gabrd | GO:0045211 | postsynaptic membrane | IEA | Inferred from Electronic Annotation | gamma-aminobutyric acid (GABA) A receptor, delta |  | gene_product | taxon:7955 | Danio rerio | ZFIN |
| Gabrg2 | GO:0045202 | synapse | IEA | Inferred from Electronic Annotation | gamma-aminobutyric acid (GABA) A receptor, subunit gamma 2 | GABAA-R|Gabrg-2|gamma2 | protein | taxon:10090 | Mus musculus | UniProtKB |
| gabrg2 | GO:0045202 | synapse | IEA | Inferred from Electronic Annotation | gamma-aminobutyric acid (GABA) A receptor, gamma 2 |  | gene_product | taxon:7955 | Danio rerio | ZFIN |
| GABRG2 | GO:0045211 | postsynaptic membrane | IEA | Inferred from Electronic Annotation | Gamma-aminobutyric acid receptor subunit gamma-2 | F1NID8_CHICK|GABRG2 | protein | taxon:9031 | Gallus gallus | UniProtKB |
| GABRG2 | GO:0045211 | postsynaptic membrane | IEA | Inferred from Electronic Annotation | Gamma-aminobutyric acid receptor subunit gamma-2 | F1NVG2_CHICK|GABRG2 | protein | taxon:9031 | Gallus gallus | UniProtKB |
| GABRG2 | GO:0045211 | postsynaptic membrane | IEA | Inferred from Electronic Annotation | Gamma-aminobutyric acid receptor subunit gamma-2 | GABRG2|GBRG2_CHICK | protein | taxon:9031 | Gallus gallus | UniProtKB |
| GABRG2 | GO:0045211 | postsynaptic membrane | IEA | Inferred from Electronic Annotation | Gamma-aminobutyric acid receptor subunit gamma-2 | F1MIG7_BOVIN|GABRG2 | protein | taxon:9913 | Bos taurus | UniProtKB |
| GABRG2 | GO:0045211 | postsynaptic membrane | IEA | Inferred from Electronic Annotation | Gamma-aminobutyric acid receptor subunit gamma-2 | G3MYR4_BOVIN|GABRG2 | protein | taxon:9913 | Bos taurus | UniProtKB |
| GABRG2 | GO:0045211 | postsynaptic membrane | IEA | Inferred from Electronic Annotation | Gamma-aminobutyric acid receptor subunit gamma-2 | GABRG2|GBRG2_BOVIN | protein | taxon:9913 | Bos taurus | UniProtKB |
| GABRG2 | GO:0045211 | postsynaptic membrane | IEA | Inferred from Electronic Annotation | Uncharacterized protein | E2RSQ0_CANFA|GABRG2 | protein | taxon:9615 | Canis familiaris | UniProtKB |
| GABRG2 | GO:0045211 | postsynaptic membrane | IEA | Inferred from Electronic Annotation | Gamma-aminobutyric acid receptor subunit gamma-2 | A8MWU7_HUMAN|GABRG2 | protein | taxon:9606 | Homo sapiens | UniProtKB |
| GABRG2 | GO:0045211 | postsynaptic membrane | IEA | Inferred from Electronic Annotation | Gamma-aminobutyric acid receptor subunit gamma-2 | E5RGG2_HUMAN|GABRG2 | protein | taxon:9606 | Homo sapiens | UniProtKB |
| GABRG2 | GO:0045211 | postsynaptic membrane | IEA | Inferred from Electronic Annotation | Gamma-aminobutyric acid receptor subunit gamma-2 | F5HB82_HUMAN|GABRG2 | protein | taxon:9606 | Homo sapiens | UniProtKB |
| GABRG2 | GO:0045211 | postsynaptic membrane | IEA | Inferred from Electronic Annotation | Gamma-aminobutyric acid receptor subunit gamma-2 | GABRG2|GBRG2_HUMAN | protein | taxon:9606 | Homo sapiens | UniProtKB |
| GABRG2 | GO:0045211 | postsynaptic membrane | IEA | Inferred from Electronic Annotation | Uncharacterized protein | F1RR72_PIG|GABRG2 | protein | taxon:9823 | Sus scrofa | UniProtKB |
| Gabrg2 | GO:0045211 | postsynaptic membrane | IEA | Inferred from Electronic Annotation | gamma-aminobutyric acid (GABA) A receptor, subunit gamma 2 | GABAA-R|Gabrg-2|gamma2 | protein | taxon:10090 | Mus musculus | UniProtKB |
| Gabrg2 | GO:0045211 | postsynaptic membrane | IEA | Inferred from Electronic Annotation | gamma-aminobutyric acid (GABA) A receptor, subunit gamma 2 | GABAA-R|Gabrg-2|gamma2 | protein | taxon:10090 | Mus musculus | UniProtKB |
| Gabrg2 | GO:0045211 | postsynaptic membrane | IEA | Inferred from Electronic Annotation | gamma-aminobutyric acid (GABA) A receptor, gamma 2 |  | gene | taxon:10116 | Rattus norvegicus | UniProtKB |
| Gabrg2 | GO:0045211 | postsynaptic membrane | IEA | Inferred from Electronic Annotation | Gamma-aminobutyric acid receptor subunit gamma-2 | F1M4V9_RAT|Gabrg2 | protein | taxon:10116 | Rattus norvegicus | UniProtKB |
| gabrg2 | GO:0045211 | postsynaptic membrane | IEA | Inferred from Electronic Annotation | gamma-aminobutyric acid (GABA) A receptor, gamma 2 |  | gene_product | taxon:7955 | Danio rerio | ZFIN |
| gabrg2 | GO:0045211 | postsynaptic membrane | IEA | Inferred from Electronic Annotation | gamma-aminobutyric acid (GABA) A receptor, gamma 2 |  | gene_product | taxon:7955 | Danio rerio | ZFIN |
| Gabrg2 | GO:0060077 | inhibitory synapse | ISO | Inferred from Sequence Orthology | gamma-aminobutyric acid (GABA) A receptor, subunit gamma 2 | GABAA-R|Gabrg-2|gamma2 | protein | taxon:10090 | Mus musculus | MGI |
| Gabrg2 | GO:0060077 | inhibitory synapse | IDA | Inferred from Direct Assay | gamma-aminobutyric acid (GABA) A receptor, gamma 2 |  | gene | taxon:10116 | Rattus norvegicus | MGI |
| Gad1 | GO:0048786 | presynaptic active zone | IDA | Inferred from Direct Assay | glutamate decarboxylase 1 | EP10|Gad-1|GAD25|GAD44|GAD67|Z49976 | protein | taxon:10090 | Mus musculus | MGI |
| Gad1 | GO:0048786 | presynaptic active zone | ISO | Inferred from Sequence Orthology | glutamate decarboxylase 1 |  | gene | taxon:10116 | Rattus norvegicus | RGD |
| Gad1 | GO:0045202 | synapse | IDA | Inferred from Direct Assay | glutamate decarboxylase 1 | EP10|Gad-1|GAD25|GAD44|GAD67|Z49976 | protein | taxon:10090 | Mus musculus | MGI |
| Gad1 | GO:0045202 | synapse | ISO | Inferred from Sequence Orthology | glutamate decarboxylase 1 |  | gene | taxon:10116 | Rattus norvegicus | RGD |
| GAD2 | GO:0030672 | synaptic vesicle membrane | IEA | Inferred from Electronic Annotation | Glutamate decarboxylase 2 | DCE2_HUMAN|GAD2|GAD65 | protein | taxon:9606 | Homo sapiens | ENSEMBL |
| Gad2 | GO:0030672 | synaptic vesicle membrane | ISO | Inferred from Sequence Orthology | glutamic acid decarboxylase 2 | GAD(65)|Gad-2|GAD65 | protein | taxon:10090 | Mus musculus | RGD |
| Gad2 | GO:0030672 | synaptic vesicle membrane | IDA | Inferred from Direct Assay | glutamate decarboxylase 2 |  | gene | taxon:10116 | Rattus norvegicus | RGD |
| GAD2 | GO:0042734 | presynaptic membrane | IEA | Inferred from Electronic Annotation | Glutamate decarboxylase 2 | DCE2_CANFA|GAD2|GAD65 | protein | taxon:9615 | Canis familiaris | UniProtKB |
| GAD2 | GO:0042734 | presynaptic membrane | IEA | Inferred from Electronic Annotation | Glutamate decarboxylase 2 | DCE2_HUMAN|GAD2|GAD65 | protein | taxon:9606 | Homo sapiens | UniProtKB |
| GAD2 | GO:0042734 | presynaptic membrane | IEA | Inferred from Electronic Annotation | Glutamate decarboxylase 2 | DCE2_PIG|GAD2|GAD65 | protein | taxon:9823 | Sus scrofa | UniProtKB |
| Gad2 | GO:0042734 | presynaptic membrane | IEA | Inferred from Electronic Annotation | glutamate decarboxylase 2 |  | gene | taxon:10116 | Rattus norvegicus | UniProtKB |
| GAD2 | GO:0045202 | synapse | IEA | Inferred from Electronic Annotation | Uncharacterized protein | F1N890_CHICK|GAD2 | protein | taxon:9031 | Gallus gallus | ENSEMBL |
| GAD2 | GO:0045202 | synapse | IEA | Inferred from Electronic Annotation | Uncharacterized protein | F1NS42_CHICK|GAD2 | protein | taxon:9031 | Gallus gallus | ENSEMBL |
| GAD2 | GO:0045202 | synapse | IEA | Inferred from Electronic Annotation | Uncharacterized protein | F1N6X2_BOVIN|GAD2 | protein | taxon:9913 | Bos taurus | ENSEMBL |
| Gad2 | GO:0045202 | synapse | IDA | Inferred from Direct Assay | glutamic acid decarboxylase 2 | GAD(65)|Gad-2|GAD65 | protein | taxon:10090 | Mus musculus | MGI |
| Gad2 | GO:0045202 | synapse | ISO | Inferred from Sequence Orthology | glutamate decarboxylase 2 |  | gene | taxon:10116 | Rattus norvegicus | RGD |
| ICA1 | GO:0030672 | synaptic vesicle membrane | IEA | Inferred from Electronic Annotation | Uncharacterized protein | E1C2A8_CHICK|ICA1 | protein | taxon:9031 | Gallus gallus | ENSEMBL |
| ICA1 | GO:0030672 | synaptic vesicle membrane | IEA | Inferred from Electronic Annotation | Islet cell autoantigen 1 | C9J3Y4_HUMAN|ICA1 | protein | taxon:9606 | Homo sapiens | ENSEMBL |
| ICA1 | GO:0030672 | synaptic vesicle membrane | IEA | Inferred from Electronic Annotation | Islet cell autoantigen 1 | F8WET5_HUMAN|ICA1 | protein | taxon:9606 | Homo sapiens | ENSEMBL |
| ICA1 | GO:0030672 | synaptic vesicle membrane | ISS | Inferred from Sequence or Structural Similarity | Islet cell autoantigen 1 | ICA1|ICA69_HUMAN | protein | taxon:9606 | Homo sapiens | UniProtKB |
| Ica1 | GO:0030672 | synaptic vesicle membrane | IDA | Inferred from Direct Assay | islet cell autoantigen 1 | 69kDa|ICA69 | protein | taxon:10090 | Mus musculus | UniProtKB |
| Ica1 | GO:0030672 | synaptic vesicle membrane | IEA | Inferred from Electronic Annotation | islet cell autoantigen 1 |  | gene | taxon:10116 | Rattus norvegicus | ENSEMBL |
| Ica1 | GO:0030672 | synaptic vesicle membrane | ISS | Inferred from Sequence or Structural Similarity | islet cell autoantigen 1 |  | gene | taxon:10116 | Rattus norvegicus | UniProtKB |
| Ica1 | GO:0030672 | synaptic vesicle membrane | ISO | Inferred from Sequence Orthology | islet cell autoantigen 1 |  | gene | taxon:10116 | Rattus norvegicus | RGD |
| Ica1 | GO:0045202 | synapse | IEA | Inferred from Electronic Annotation | islet cell autoantigen 1 | 69kDa|ICA69 | protein | taxon:10090 | Mus musculus | UniProtKB |
| ITPR1 | GO:0014069 | postsynaptic density | IEA | Inferred from Electronic Annotation | Uncharacterized protein | F1P1N6_CHICK|ITPR1 | protein | taxon:9031 | Gallus gallus | ENSEMBL |
| ITPR1 | GO:0014069 | postsynaptic density | IEA | Inferred from Electronic Annotation | Uncharacterized protein | ITPR1|Q9DEX2_CHICK | protein | taxon:9031 | Gallus gallus | ENSEMBL |
| ITPR1 | GO:0014069 | postsynaptic density | IEA | Inferred from Electronic Annotation | Uncharacterized protein | F1PIQ7_CANFA|ITPR1 | protein | taxon:9615 | Canis familiaris | ENSEMBL |
| ITPR1 | GO:0014069 | postsynaptic density | IEA | Inferred from Electronic Annotation | Uncharacterized protein | F1PW86_CANFA|ITPR1 | protein | taxon:9615 | Canis familiaris | ENSEMBL |
| ITPR1 | GO:0014069 | postsynaptic density | IEA | Inferred from Electronic Annotation | Inositol 1,4,5-trisphosphate receptor type 1 | E7EPX7_HUMAN|ITPR1 | protein | taxon:9606 | Homo sapiens | ENSEMBL |
| Itpr1 | GO:0014069 | postsynaptic density | IDA | Inferred from Direct Assay | inositol 1,4,5-trisphosphate receptor 1 | InsP3R type I|Ip3r|IP3R1|Itpr-1|opt|P400|Pcp-1|Pcp1 | protein | taxon:10090 | Mus musculus | MGI |
| Itpr1 | GO:0014069 | postsynaptic density | ISO | Inferred from Sequence Orthology | inositol 1,4,5-trisphosphate receptor 1 | InsP3R type I|Ip3r|IP3R1|Itpr-1|opt|P400|Pcp-1|Pcp1 | protein | taxon:10090 | Mus musculus | RGD |
| Itpr1 | GO:0014069 | postsynaptic density | IDA | Inferred from Direct Assay | inositol 1,4,5-trisphosphate receptor, type 1 |  | gene | taxon:10116 | Rattus norvegicus | RGD |
| Itpr1 | GO:0014069 | postsynaptic density | ISO | Inferred from Sequence Orthology | inositol 1,4,5-trisphosphate receptor, type 1 |  | gene | taxon:10116 | Rattus norvegicus | RGD |
| Itpr1 | GO:0014069 | postsynaptic density | IEA | Inferred from Electronic Annotation | Inositol 1,4,5-trisphosphate receptor type 1 | F1LSF7_RAT|Itpr1 | protein | taxon:10116 | Rattus norvegicus | ENSEMBL |
| Itpr1 | GO:0097060 | synaptic membrane | ISO | Inferred from Sequence Orthology | inositol 1,4,5-trisphosphate receptor 1 | InsP3R type I|Ip3r|IP3R1|Itpr-1|opt|P400|Pcp-1|Pcp1 | protein | taxon:10090 | Mus musculus | RGD |
| Itpr1 | GO:0097060 | synaptic membrane | IDA | Inferred from Direct Assay | inositol 1,4,5-trisphosphate receptor, type 1 |  | gene | taxon:10116 | Rattus norvegicus | RGD |
| PTK2B | GO:0014069 | postsynaptic density | IEA | Inferred from Electronic Annotation | Protein-tyrosine kinase 2-beta | C9JHV9_HUMAN|PTK2B | protein | taxon:9606 | Homo sapiens | ENSEMBL |
| PTK2B | GO:0014069 | postsynaptic density | IEA | Inferred from Electronic Annotation | Protein-tyrosine kinase 2-beta | E5RHL2_HUMAN|PTK2B | protein | taxon:9606 | Homo sapiens | ENSEMBL |
| PTK2B | GO:0014069 | postsynaptic density | IEA | Inferred from Electronic Annotation | Protein-tyrosine kinase 2-beta | E5RJ77_HUMAN|PTK2B | protein | taxon:9606 | Homo sapiens | ENSEMBL |
| PTK2B | GO:0014069 | postsynaptic density | IEA | Inferred from Electronic Annotation | Protein-tyrosine kinase 2-beta | E5RK84_HUMAN|PTK2B | protein | taxon:9606 | Homo sapiens | ENSEMBL |
| PTK2B | GO:0014069 | postsynaptic density | IEA | Inferred from Electronic Annotation | Protein-tyrosine kinase 2-beta | FAK2|FAK2_HUMAN|PTK2B|PYK2|RAFTK | protein | taxon:9606 | Homo sapiens | ENSEMBL |
| Ptk2b | GO:0014069 | postsynaptic density | ISO | Inferred from Sequence Orthology | PTK2 protein tyrosine kinase 2 beta | CAKbeta|calcium-dependent tyrosine kinase|cellular adhesion kinase beta|proline-rich tyrosine kinase 2|PYK2|Raftk|related adhesion focal tyrosine kinase | protein | taxon:10090 | Mus musculus | RGD |
| Ptk2b | GO:0014069 | postsynaptic density | IDA | Inferred from Direct Assay | PTK2B protein tyrosine kinase 2 beta |  | gene | taxon:10116 | Rattus norvegicus | RGD |
| SLC32A1 | GO:0030672 | synaptic vesicle membrane | TAS | Traceable Author Statement | Vesicular inhibitory amino acid transporter | SLC32A1|VGAT|VIAAT|VIAAT_HUMAN | protein | taxon:9606 | Homo sapiens | Reactome |
| Slc32a1 | GO:0045202 | synapse | ISO | Inferred from Sequence Orthology | solute carrier family 32 (GABA vesicular transporter), member 1 | R75019|VGAT|Viaat | protein | taxon:10090 | Mus musculus | MGI |
| Slc32a1 | GO:0045202 | synapse | IDA | Inferred from Direct Assay | solute carrier family 32 (GABA vesicular transporter), member 1 |  | gene | taxon:10116 | Rattus norvegicus | MGI |
| Slc32a1 | GO:0008021 | synaptic vesicle | ISO | Inferred from Sequence Orthology | solute carrier family 32 (GABA vesicular transporter), member 1 | R75019|VGAT|Viaat | protein | taxon:10090 | Mus musculus | RGD |
| Slc32a1 | GO:0008021 | synaptic vesicle | IDA | Inferred from Direct Assay | solute carrier family 32 (GABA vesicular transporter), member 1 |  | gene | taxon:10116 | Rattus norvegicus | RGD |
| SLC32A1 | GO:0060077 | inhibitory synapse | IEA | Inferred from Electronic Annotation | Uncharacterized protein | F1NLZ7_CHICK|SLC32A1 | protein | taxon:9031 | Gallus gallus | ENSEMBL |
| SLC32A1 | GO:0060077 | inhibitory synapse | IEA | Inferred from Electronic Annotation | Uncharacterized protein | G3N354_BOVIN|SLC32A1 | protein | taxon:9913 | Bos taurus | ENSEMBL |
| SLC32A1 | GO:0060077 | inhibitory synapse | IEA | Inferred from Electronic Annotation | Uncharacterized protein | E2QU68_CANFA|SLC32A1 | protein | taxon:9615 | Canis familiaris | ENSEMBL |
| SLC32A1 | GO:0060077 | inhibitory synapse | IEA | Inferred from Electronic Annotation | Vesicular inhibitory amino acid transporter | SLC32A1|VGAT|VIAAT|VIAAT_HUMAN | protein | taxon:9606 | Homo sapiens | ENSEMBL |
| SLC32A1 | GO:0060077 | inhibitory synapse | IEA | Inferred from Electronic Annotation | Uncharacterized protein | F1SDX2_PIG|SLC32A1 | protein | taxon:9823 | Sus scrofa | ENSEMBL |
| Slc32a1 | GO:0060077 | inhibitory synapse | IDA | Inferred from Direct Assay | solute carrier family 32 (GABA vesicular transporter), member 1 | R75019|VGAT|Viaat | protein | taxon:10090 | Mus musculus | MGI |
| Slc32a1 | GO:0060077 | inhibitory synapse | IDA | Inferred from Direct Assay | solute carrier family 32 (GABA vesicular transporter), member 1 | R75019|VGAT|Viaat | protein | taxon:10090 | Mus musculus | MGI |
| Slc32a1 | GO:0060077 | inhibitory synapse | ISO | Inferred from Sequence Orthology | solute carrier family 32 (GABA vesicular transporter), member 1 | R75019|VGAT|Viaat | protein | taxon:10090 | Mus musculus | RGD |
| Slc32a1 | GO:0060077 | inhibitory synapse | IDA | Inferred from Direct Assay | solute carrier family 32 (GABA vesicular transporter), member 1 |  | gene | taxon:10116 | Rattus norvegicus | BHF-UCL |
| Slc32a1 | GO:0060077 | inhibitory synapse | ISO | Inferred from Sequence Orthology | solute carrier family 32 (GABA vesicular transporter), member 1 |  | gene | taxon:10116 | Rattus norvegicus | RGD |
| SVOP | GO:0030672 | synaptic vesicle membrane | IEA | Inferred from Electronic Annotation | Synaptic vesicle 2-related protein | SVOP|SVOP_BOVIN | protein | taxon:9913 | Bos taurus | UniProtKB |
| SVOP | GO:0030672 | synaptic vesicle membrane | IEA | Inferred from Electronic Annotation | Synaptic vesicle 2-related protein | SVOP|SVOP_HUMAN | protein | taxon:9606 | Homo sapiens | UniProtKB |
| Svop | GO:0030672 | synaptic vesicle membrane | IEA | Inferred from Electronic Annotation | SV2 related protein |  | gene | taxon:10116 | Rattus norvegicus | UniProtKB |
| Svop | GO:0045202 | synapse | IEA | Inferred from Electronic Annotation | SV2 related protein | msvop | protein | taxon:10090 | Mus musculus | UniProtKB |
| SVOP | GO:0008021 | synaptic vesicle | ISS | Inferred from Sequence or Structural Similarity | Synaptic vesicle 2-related protein | SVOP|SVOP_HUMAN | protein | taxon:9606 | Homo sapiens | HGNC |
| Svop | GO:0008021 | synaptic vesicle | ISO | Inferred from Sequence Orthology | SV2 related protein | msvop | protein | taxon:10090 | Mus musculus | RGD |
| Svop | GO:0008021 | synaptic vesicle | IDA | Inferred from Direct Assay | SV2 related protein |  | gene | taxon:10116 | Rattus norvegicus | HGNC |
| Syn2 | GO:0030672 | synaptic vesicle membrane | IDA | Inferred from Direct Assay | synapsin II | Synapsin IIa|Synapsin IIb | protein | taxon:10090 | Mus musculus | MGI |
| Syn2 | GO:0030672 | synaptic vesicle membrane | IEA | Inferred from Electronic Annotation | synapsin II |  | gene | taxon:10116 | Rattus norvegicus | ENSEMBL |
| Syn2 | GO:0030672 | synaptic vesicle membrane | ISO | Inferred from Sequence Orthology | synapsin II |  | gene | taxon:10116 | Rattus norvegicus | RGD |
| Syn2 | GO:0045202 | synapse | IDA | Inferred from Direct Assay | synapsin II | Synapsin IIa|Synapsin IIb | protein | taxon:10090 | Mus musculus | MGI |
| Syn2 | GO:0045202 | synapse | ISO | Inferred from Sequence Orthology | synapsin II |  | gene | taxon:10116 | Rattus norvegicus | RGD |
| SYN2 | GO:0008021 | synaptic vesicle | IEA | Inferred from Electronic Annotation | Synapsin-2 | F5H7H8_HUMAN|SYN2 | protein | taxon:9606 | Homo sapiens | InterPro |
| SYN2 | GO:0008021 | synaptic vesicle | IEA | Inferred from Electronic Annotation | Synapsin-2 | SYN2|SYN2_HUMAN | protein | taxon:9606 | Homo sapiens | InterPro |
| Syn2 | GO:0008021 | synaptic vesicle | ISO | Inferred from Sequence Orthology | synapsin II | Synapsin IIa|Synapsin IIb | protein | taxon:10090 | Mus musculus | RGD |
| Syn2 | GO:0008021 | synaptic vesicle | IDA | Inferred from Direct Assay | synapsin II |  | gene | taxon:10116 | Rattus norvegicus | UniProtKB |
| Syn2 | GO:0008021 | synaptic vesicle | IEA | Inferred from Electronic Annotation | synapsin II |  | gene | taxon:10116 | Rattus norvegicus | InterPro |
| Syn2 | GO:0008021 | synaptic vesicle | TAS | Traceable Author Statement | synapsin II |  | gene | taxon:10116 | Rattus norvegicus | RGD |
| SYP | GO:0048786 | presynaptic active zone | IEA | Inferred from Electronic Annotation | Synaptophysin | F1N548_BOVIN|SYP | protein | taxon:9913 | Bos taurus | ENSEMBL |
| SYP | GO:0048786 | presynaptic active zone | IEA | Inferred from Electronic Annotation | cDNA, FLJ78852, highly similar to Synaptophysin | B7Z359_HUMAN|SYP | protein | taxon:9606 | Homo sapiens | ENSEMBL |
| SYP | GO:0048786 | presynaptic active zone | IEA | Inferred from Electronic Annotation | Synaptophysin | SYP|SYPH_HUMAN | protein | taxon:9606 | Homo sapiens | ENSEMBL |
| SYP | GO:0048786 | presynaptic active zone | IEA | Inferred from Electronic Annotation | Uncharacterized protein | F1RW46_PIG|SYP | protein | taxon:9823 | Sus scrofa | ENSEMBL |
| Syp | GO:0048786 | presynaptic active zone | IDA | Inferred from Direct Assay | synaptophysin | p38|Syn|Syp I | protein | taxon:10090 | Mus musculus | BHF-UCL |
| Syp | GO:0048786 | presynaptic active zone | IEA | Inferred from Electronic Annotation | synaptophysin |  | gene | taxon:10116 | Rattus norvegicus | ENSEMBL |
| Syp | GO:0048786 | presynaptic active zone | ISO | Inferred from Sequence Orthology | synaptophysin |  | gene | taxon:10116 | Rattus norvegicus | RGD |
| Syp | GO:0060076 | excitatory synapse | ISO | Inferred from Sequence Orthology | synaptophysin | p38|Syn|Syp I | protein | taxon:10090 | Mus musculus | RGD |
| Syp | GO:0060076 | excitatory synapse | IDA | Inferred from Direct Assay | synaptophysin |  | gene | taxon:10116 | Rattus norvegicus | BHF-UCL |
| SYP | GO:0030672 | synaptic vesicle membrane | IEA | Inferred from Electronic Annotation | Synaptophysin | F1N548_BOVIN|SYP | protein | taxon:9913 | Bos taurus | ENSEMBL |
| SYP | GO:0030672 | synaptic vesicle membrane | IEA | Inferred from Electronic Annotation | Synaptophysin | SYP|SYPH_BOVIN | protein | taxon:9913 | Bos taurus | UniProtKB |
| SYP | GO:0030672 | synaptic vesicle membrane | IEA | Inferred from Electronic Annotation | cDNA, FLJ78852, highly similar to Synaptophysin | B7Z359_HUMAN|SYP | protein | taxon:9606 | Homo sapiens | ENSEMBL |
| SYP | GO:0030672 | synaptic vesicle membrane | IEA | Inferred from Electronic Annotation | Uncharacterized protein | F1RW46_PIG|SYP | protein | taxon:9823 | Sus scrofa | ENSEMBL |
| Syp | GO:0030672 | synaptic vesicle membrane | IDA | Inferred from Direct Assay | synaptophysin | p38|Syn|Syp I | protein | taxon:10090 | Mus musculus | MGI |
| Syp | GO:0030672 | synaptic vesicle membrane | IDA | Inferred from Direct Assay | synaptophysin | p38|Syn|Syp I | protein | taxon:10090 | Mus musculus | MGI |
| Syp | GO:0030672 | synaptic vesicle membrane | ISO | Inferred from Sequence Orthology | synaptophysin |  | gene | taxon:10116 | Rattus norvegicus | RGD |
| SYP | GO:0042734 | presynaptic membrane | IEA | Inferred from Electronic Annotation | Synaptophysin | F1N548_BOVIN|SYP | protein | taxon:9913 | Bos taurus | ENSEMBL |
| SYP | GO:0042734 | presynaptic membrane | IEA | Inferred from Electronic Annotation | cDNA, FLJ78852, highly similar to Synaptophysin | B7Z359_HUMAN|SYP | protein | taxon:9606 | Homo sapiens | ENSEMBL |
| SYP | GO:0042734 | presynaptic membrane | IEA | Inferred from Electronic Annotation | Synaptophysin | SYP|SYPH_HUMAN | protein | taxon:9606 | Homo sapiens | ENSEMBL |
| SYP | GO:0042734 | presynaptic membrane | IEA | Inferred from Electronic Annotation | Uncharacterized protein | F1RW46_PIG|SYP | protein | taxon:9823 | Sus scrofa | ENSEMBL |
| Syp | GO:0042734 | presynaptic membrane | IDA | Inferred from Direct Assay | synaptophysin | p38|Syn|Syp I | protein | taxon:10090 | Mus musculus | MGI |
| Syp | GO:0042734 | presynaptic membrane | IDA | Inferred from Direct Assay | synaptophysin | p38|Syn|Syp I | protein | taxon:10090 | Mus musculus | UniProtKB |
| Syp | GO:0042734 | presynaptic membrane | IEA | Inferred from Electronic Annotation | synaptophysin |  | gene | taxon:10116 | Rattus norvegicus | ENSEMBL |
| Syp | GO:0042734 | presynaptic membrane | ISO | Inferred from Sequence Orthology | synaptophysin |  | gene | taxon:10116 | Rattus norvegicus | RGD |
| Syp | GO:0045202 | synapse | IDA | Inferred from Direct Assay | synaptophysin | p38|Syn|Syp I | protein | taxon:10090 | Mus musculus | MGI |
| Syp | GO:0045202 | synapse | IDA | Inferred from Direct Assay | synaptophysin | p38|Syn|Syp I | protein | taxon:10090 | Mus musculus | MGI |
| Syp | GO:0045202 | synapse | IDA | Inferred from Direct Assay | synaptophysin | p38|Syn|Syp I | protein | taxon:10090 | Mus musculus | MGI |
| Syp | GO:0045202 | synapse | IDA | Inferred from Direct Assay | synaptophysin | p38|Syn|Syp I | protein | taxon:10090 | Mus musculus | MGI |
| Syp | GO:0045202 | synapse | IDA | Inferred from Direct Assay | synaptophysin | p38|Syn|Syp I | protein | taxon:10090 | Mus musculus | MGI |
| Syp | GO:0045202 | synapse | IDA | Inferred from Direct Assay | synaptophysin | p38|Syn|Syp I | protein | taxon:10090 | Mus musculus | MGI |
| Syp | GO:0045202 | synapse | IDA | Inferred from Direct Assay | synaptophysin | p38|Syn|Syp I | protein | taxon:10090 | Mus musculus | MGI |
| Syp | GO:0045202 | synapse | IDA | Inferred from Direct Assay | synaptophysin | p38|Syn|Syp I | protein | taxon:10090 | Mus musculus | MGI |
| Syp | GO:0045202 | synapse | ISO | Inferred from Sequence Orthology | synaptophysin |  | gene | taxon:10116 | Rattus norvegicus | RGD |
| Syp | GO:0045202 | synapse | ISO | Inferred from Sequence Orthology | synaptophysin |  | gene | taxon:10116 | Rattus norvegicus | RGD |
| SYP | GO:0008021 | synaptic vesicle | IEA | Inferred from Electronic Annotation | Uncharacterized protein | F1Q4G9_CANFA|SYP | protein | taxon:9615 | Canis familiaris | InterPro |
| SYP | GO:0008021 | synaptic vesicle | IEA | Inferred from Electronic Annotation | Synaptophysin | F2Z3E1_HUMAN|SYP | protein | taxon:9606 | Homo sapiens | InterPro |
| SYP | GO:0008021 | synaptic vesicle | IEA | Inferred from Electronic Annotation | Synaptophysin, isoform CRA_a | G5E9A2_HUMAN|hCG_2039818|SYP | protein | taxon:9606 | Homo sapiens | InterPro |
| SYP | GO:0008021 | synaptic vesicle | IEA | Inferred from Electronic Annotation | Synaptophysin | H7C4W3_HUMAN|SYP | protein | taxon:9606 | Homo sapiens | InterPro |
| Syp | GO:0008021 | synaptic vesicle | IDA | Inferred from Direct Assay | synaptophysin | p38|Syn|Syp I | protein | taxon:10090 | Mus musculus | MGI |
| Syp | GO:0008021 | synaptic vesicle | IDA | Inferred from Direct Assay | synaptophysin | p38|Syn|Syp I | protein | taxon:10090 | Mus musculus | MGI |
| Syp | GO:0008021 | synaptic vesicle | IDA | Inferred from Direct Assay | synaptophysin | p38|Syn|Syp I | protein | taxon:10090 | Mus musculus | MGI |
| Syp | GO:0008021 | synaptic vesicle | IDA | Inferred from Direct Assay | synaptophysin | p38|Syn|Syp I | protein | taxon:10090 | Mus musculus | MGI |
| Syp | GO:0008021 | synaptic vesicle | ISO | Inferred from Sequence Orthology | synaptophysin | p38|Syn|Syp I | protein | taxon:10090 | Mus musculus | MGI |
| Syp | GO:0008021 | synaptic vesicle | IDA | Inferred from Direct Assay | synaptophysin | p38|Syn|Syp I | protein | taxon:10090 | Mus musculus | MGI |
| Syp | GO:0008021 | synaptic vesicle | IDA | Inferred from Direct Assay | synaptophysin |  | gene | taxon:10116 | Rattus norvegicus | MGI |
| Syp | GO:0008021 | synaptic vesicle | IEA | Inferred from Electronic Annotation | synaptophysin |  | gene | taxon:10116 | Rattus norvegicus | InterPro |
| Syp | GO:0008021 | synaptic vesicle | ISO | Inferred from Sequence Orthology | synaptophysin |  | gene | taxon:10116 | Rattus norvegicus | RGD |
| Syp | GO:0008021 | synaptic vesicle | ISO | Inferred from Sequence Orthology | synaptophysin |  | gene | taxon:10116 | Rattus norvegicus | RGD |
| SYP | GO:0030285 | integral to synaptic vesicle membrane | NAS | Non-traceable Author Statement | Synaptophysin | SYP|SYPH_HUMAN | protein | taxon:9606 | Homo sapiens | UniProtKB |
| Syp | GO:0030285 | integral to synaptic vesicle membrane | TAS | Traceable Author Statement | synaptophysin | p38|Syn|Syp I | protein | taxon:10090 | Mus musculus | MGI |
| Syp | GO:0030285 | integral to synaptic vesicle membrane | TAS | Traceable Author Statement | synaptophysin |  | gene | taxon:10116 | Rattus norvegicus | RGD |
| VAMP1 | GO:0030672 | synaptic vesicle membrane | IEA | Inferred from Electronic Annotation | Vesicle-associated membrane protein 1 | SYB1|VAMP1|VAMP1_BOVIN | protein | taxon:9913 | Bos taurus | UniProtKB |
| VAMP1 | GO:0030672 | synaptic vesicle membrane | IEA | Inferred from Electronic Annotation | Vesicle-associated membrane protein 1 | SYB1|VAMP1|VAMP1_HUMAN | protein | taxon:9606 | Homo sapiens | UniProtKB |
| Vamp1 | GO:0030672 | synaptic vesicle membrane | IEA | Inferred from Electronic Annotation |  |  | gene | taxon:10116 | Rattus norvegicus | UniProtKB |
| Vamp1 | GO:0045202 | synapse | IEA | Inferred from Electronic Annotation |  | lew|Syb-1|Syb1|VAMP-1 | protein | taxon:10090 | Mus musculus | UniProtKB |
