# Supplementary material for: Genes for endosomal NHE6 and NHE9 are misregulated in autism brains
Source: Mol Psychiatry. 2013 Mar 19;19(3):277–9. doi: 10.1038/mp.2013.28 (PMC3932404; doi:10.1038/mp.2013.28)
Supplement: Supplementary Table 3 [file mp201328x8.doc]

**Supplementary Table 3.** Analysis of gene expression changes for NHE family comparing postmortem autism cortex to control.

| Gene | P-value | Fold change in autism |
| --- | --- | --- |
| **NHE1** | **0.0030** | **0.83** |
| NHE2 | 0.49 | 1.03 |
| NHE3 | 0.34 | 0.97 |
| NHE4 | 0.52 | 1.01 |
| NHE5 | 0.65 | 0.97 |
| **NHE6** | **0.0042** | **0.81** |
| NHE7 | 0.17 | 1.03 |
| NHE8 | 0.45 | 0.97 |
| **NHE9** | **0.00075** | **1.30** |
| NHE10 | 0.39 | 1.02 |
| NHE11 | 0.62 | 0.99 |
