# Supplementary material for: Genes for endosomal NHE6 and NHE9 are misregulated in autism brains
Source: Mol Psychiatry. 2013 Mar 19;19(3):277–9. doi: 10.1038/mp.2013.28 (PMC3932404; doi:10.1038/mp.2013.28)
Supplement: Supplementary Table 4 [file mp201328x9.doc]

**Supplementary Table 4**

| Dataset | Voineagu | Chow | Garbett |
| --- | --- | --- | --- |
| Sample size | Autism: 29 samples (16 frontal cortex, 13 temporal cortex; 16 total cases)  Control: 29 samples (16 frontal cortex, 13 temporal cortex; 16 total cases) | Autism: 15 samples (9 were ≤14y, 6 were ≥15y)  Control: 18 samples (7 were ≤14y, 11 were ≥15y) | Autism: 6 samples  Control: 6 samples |
| Brain region | Superior temporal gyrus, prefrontal cortex | Dorsolateral prefrontal cortex | Superior temporal gyrus |
| Age range | Autism: 5-51 Control: 16-56 | Autism: 2-51 Control: 4-56 | Autism: 4-30 Control: 4-30 |
| Gender | Autism: 7F, 22M Control: 2F, 27M | Autism: 15M Control: 18M | Autism: 4M, 2F Control: 4M, 2F |
| Post-mortem interval range | 4.75-43.25 hours | 4-43.25 hours | 8.3-24.0 hours |
| Source of cases | Autism Tissue Project and the Harvard brain bank | National Institute of Child Health and Human Development, University of Maryland brain bank, and the Autism Tissue Program | Autism Tissue Program |
