# Supplementary material for: Genes for endosomal NHE6 and NHE9 are misregulated in autism brains
Source: Mol Psychiatry. 2013 Mar 19;19(3):277–9. doi: 10.1038/mp.2013.28 (PMC3932404; doi:10.1038/mp.2013.28)
Supplement: Supplementary Information [file mp201328x10.doc]

**Supplementary Material**

**Methods.** All statistical analyses were performed using R version 2.15.0. We used three microarray datasets for analyses comparing gene expression in autism and control cortex (Supplementary Table 4). These were from: Voineagu et al. (n = 58), Chow et al. (n = 33), and Garbett et al. (n = 12). We downloaded the Voineagu et al. dataset (GSE28521) from GEO in both its normalized form and in its non-normalized form . We then re-normalized the non-normalized Voineagu et al. dataset with a log-base 2 transformation and quantile normalization using the Bioconductor package lumi. We did this re-normalization because in their analyses, Voineagu et al. excluded several *NHE* genes (specifically *NHE2*, *NHE3*, *NHE4*, *NHE7*, *NHE10*, and *NHE11*) due to low expression, and we wished to include these in exploratory analysis of the *NHE* genes. The Chow et al. dataset was downloaded from GEO (GSE28475) in its normalized format, and the Garbett et al. data was provided by the authors in its normalized format.

We performed genome-wide differential gene expression analysis of the Voineagu et al. dataset using the function *eBayes* from the Bioconductor package limma. We applied a Benjamini-Hochberg correction to the p-values, and selected cutoffs of < 0.05 of the p-value, and > 1.3 for log fold change. These cutoffs had been previously used by Voineagu et al. to identify differentially expressed genes , and the analysis discovered 392 differentially expressed genes. We then applied DAVID analysis to the 197 genes that were significantly down-regulated in autism cortex. The most significant gene set in the top scoring DAVID cluster was the Gene Ontology term “synapse,” for which the overlap consisted of 21 genes: *AMPH*, *APP*, *ATP6V0D1*, *CABP1*, *CADPS*, *CADPS2*, *CBLN4*, *CHRM1*, *GABRA1*, *GABRD*, *GABRG2*, *GAD1*, *GAD2*, *ICA1*, *ITPR1*, *PTK2B*, *SLC32A1*, *SVOP*, *SYN2*, *SYP*, and *VAMP1* (Supplementary Table 1 and Supplementary Table 2). The average expression of these synapse genes was then used for comparison with *NHE* gene expression. Differential gene expression analysis for the *NHE* genes was performed using an unpaired *t*-test assuming unequal variance.

To examine the expression of *NHE6* and *NHE9* across development, we used the BrainSpan RNA-seq dataset normalized to genes, made available by the Allen Institute . The dataset contained brain from both male and female subjects whose ages varied from age 8 weeks post-conception until 40 years old. Using this data, both *NHE6* and *NHE9* were plotted using the sixteen regions of brain that were present in both fetal and non-fetal brain. We then used R package made4 to cluster the log(RPKM + 1) expression of *NHE6* and *NHE9* with that of the other synapse genes.

**References for the Supplementary Material**

**Supplementary Tables**

**Supplementary Table 1.** Genome-wide re-analysis identified 21 synapse genes to be down-regulated.

| Gene | P-value | Fold change in autism |
| --- | --- | --- |
| AMPH | 0.029 | 0.67 |
| APP | 0.047 | 0.77 |
| ATP6V0D1 | 0.017 | 0.75 |
| CABP1 | 0.0037 | 0.72 |
| CADPS | 0.00050 | 0.72 |
| CADPS2 | 0.0015 | 0.65 |
| CBLN4 | 0.047 | 0.72 |
| CHRM1 | 0.0017 | 0.72 |
| GABRA1 | 0.023 | 0.63 |
| GABRD | 0.0063 | 0.77 |
| GABRG2 | 0.024 | 0.62 |
| GAD1 | 0.010 | 0.58 |
| GAD2 | 0.012 | 0.55 |
| ICA1 | 0.00065 | 0.72 |
| ITPR1 | 0.019 | 0.72 |
| PTK2B | 0.017 | 0.73 |
| SLC32A1 | 0.032 | 0.58 |
| SVOP | 0.0089 | 0.70 |
| SYN2 | 0.032 | 0.76 |
| SYP | 0.017 | 0.76 |
| VAMP1 | 0.00074 | 0.54 |

**Supplementary Table 2.** Evidence for and source of the relationships between each synapse-related gene and Gene Ontology term “synapse” and it children terms.

See Excel file attached.

**Supplementary Table 3.** Analysis of gene expression changes for NHE family comparing postmortem autism cortex to control.

| Gene | P-value | Fold change in autism |
| --- | --- | --- |
| **NHE1** | **0.0030** | **0.83** |
| NHE2 | 0.49 | 1.03 |
| NHE3 | 0.34 | 0.97 |
| NHE4 | 0.52 | 1.01 |
| NHE5 | 0.65 | 0.97 |
| **NHE6** | **0.0042** | **0.81** |
| NHE7 | 0.17 | 1.03 |
| NHE8 | 0.45 | 0.97 |
| **NHE9** | **0.00075** | **1.30** |
| NHE10 | 0.39 | 1.02 |
| NHE11 | 0.62 | 0.99 |

**Supplementary Table 4.** Summary of the samples from each microarray dataset, including sample size, brain region, source of tissue, and distributions of age, gender, and post-mortem interval.

See Excel file attached.

**Supplementary Figures Legends**

**Supplementary Figure 1.** NHE6 plotted across all tissues and time in Allen Institute BrainSpan RNA-seq data normalized to genes. pcw=weeks post-conception, mos=postnatal months.

**Supplementary Figure 2.** NHE9 plotted across all tissues and time in Allen Institute BrainSpan RNA-seq data normalized to genes. pcw=weeks post-conception, mos=postnatal months.

**Supplementary Figure 3.** Heatmap of NHE6 and NHE9 clustered with synapse-related genes in log(RPKM + 1) data from Allen Institute BrainSpan RNA-seq data normalized to genes. pcw=weeks post-conception, mos=postnatal months.

**Supplementary Figure 4.** NHE6 plotted against known autism-associtaed genes in the Voineagu et al. dataset.

**Supplementary Figure 5.** NHE9 plotted against known autism-associtaed genes in the Voineagu et al. dataset.
